# From disgusting and complicated to simple and brilliant: Implementation perspectives and lessons learned from users and rejectors of mail-in SARS-CoV-2 gargle tests

**DOI:** 10.3389/fpubh.2022.1024525

**Published:** 2023-01-05

**Authors:** Freda Röhr, Ferdinand Uellner, Andreas Deckert, Simon Anders, Robin Burk, Michael Knop, Lucia Brugnara, Till Bärnighausen, Albrecht Jahn, Shannon McMahon, Aurélia Souares

**Affiliations:** ^1^Heidelberg Institute of Global Health (HIGH), Faculty of Medicine and University Hospital, University of Heidelberg, Heidelberg, Germany; ^2^Center for Molecular Biology Heidelberg (ZMBH), University of Heidelberg, Heidelberg, Germany; ^3^Bioquant Center, University of Heidelberg, Heidelberg, Germany; ^4^German Cancer Research Center (DKFZ)-ZMBH Alliance, Heidelberg, Germany; ^5^Evaplan Ltd. at the University Hospital Heidelberg, Heidelberg, Germany; ^6^International Health Department, Johns Hopkins Bloomberg School of Public Health, Baltimore, MD, United States; ^7^German Center for Infection Research Heidelberg Site, Heidelberg, Germany

**Keywords:** SARS-CoV-2, mail-in tests, gargle test, self-sampling, COVID-19, test user perspectives, test rejector perspectives, implementation study

## Abstract

**Background:**

Despite the important role of testing as a measure against the COVID-19 pandemic, user perspectives on SARS-CoV-2 tests remain scarce, inhibiting an improvement of testing approaches. As the world enters the third year of the pandemic, more nuanced perspectives of testing, and opportunities to expand testing in a feasible and affordable manner merit consideration.

**Methods:**

Conducted amid the second pandemic wave (late 2020–early 2021) during and after a multi-arm trial evaluating SARS-CoV-2 surveillance strategies in the federal state Baden-Württemberg, Germany, this qualitative sub-study aimed to gain a deeper understanding of how test users and test rejectors perceived mail-in SARS-CoV-2 gargle tests. We conducted 67 semi-structured in-depth interviews (mean duration: 60 min) *via* telephone or video call. Interviews were audio-recorded, transcribed verbatim and analyzed inductively using thematic analysis. The *Consolidated Framework for Implementation Research* guided the findings' presentation.

**Results:**

Respondents generally described gargle sampling as simple and comfortable. However, individual perceptions of the testing method and its feasibility varied widely from disgusting and complicated to simple and brilliant. Self-sampling was appreciated for lowering infection risks during testing, but also considered more complex. Gargle-sampling increased participants' self-efficacy to sample correctly. Communication (first contact, quantity and content of information, reminders, support system) and trust (in the study, its institutional affiliation and test method) decisively influenced the intervention's acceptability.

**Conclusion:**

User-driven insights on how to streamline testing include: consider communication, first impressions of tests and information as key for successful mail-in testing; pay attention to the role of mutual trust between those taking and administering tests; implement gargle self-sampling as a pleasant alternative to swab testing; offer multiple test methods to increase test up-take.

## Introduction

Testing is one of the key strategies against the SARS-CoV-2 pandemic as it enables timely detection and treatment of infections and facilitates the interruption of infection chains (1). Meanwhile, vaccines have become widely available and have proven to be effective at preventing symptomatic diseases and COVID-19 related hospitalizations and mortality (2). Nevertheless, in light of unsatisfying vaccination rates, breakthrough infections, the limited duration of vaccination protection and the need to identify and monitor variants of concern, testing remains indispensable (1, 3). There is, at present, relatively limited evidence to guide countries on how to broach testing, and, partly due to this, testing approaches vary widely across countries. Recent data demonstrates a variety of testing strategies around the world, and highly variable testing rates (4). Since June 2021, the WHO recommends testing individuals that are suspected of having COVID-19, regardless of vaccination status or disease history (1). According to the WHO, asymptomatic testing should only focus on specific groups including individuals frequently exposed to SARS-CoV-2 (1). Many countries including China, Vietnam, Iceland, Germany, and Slovakia have, nonetheless, expanded their testing to widespread screening of asymptomatic individuals to shorten quarantine, protect people in high-risk settings, enable cluster response testing or increase social and economic activity (5). Some countries such as the UK introduced SARS-CoV-2 active surveillance strategies that aim at testing sufficient individuals to monitor outbreaks of disease and characterize the SARS-CoV-2 prevalence (6). Such active surveillance strategies rely on high response rates to estimate a representative prevalence and, hence, on testing being convenient and adapted to user preferences.

The first diagnostic tests, which became the gold-standard to affirm SARS-CoV-2 infections, were based on (naso-)pharyngeal swab sampling and the detection of viral nucleic acids (reverse transcription-polymerase chain reaction, RT-PCR) (1). Further analysis methods were developed including reverse transcription loop-mediated isothermal amplification (RT-LAMP) and rapid diagnostic tests (RDTs) detecting host antibodies and viral antigens (7). Sampling was supplemented by nasal and mouth swabs, gargling, or collecting saliva *via* drooling or spitting (8).

The development of innovative test methods including self-testing and self-sampling was encouraged to reduce infection risks of testing and costs by requiring fewer material and staff resources, to scale up testing efficiency and accessibility (9, 10). Critics contend that self-testing *via* RDTs came at the expense of lower and varying test accuracies and point to partly poor qualities of test centers administering RDTs (11, 12). Self-sampling has been used successfully related to HIV and other sexually transmitted infections where it has shown to be efficacious while requiring fewer resources (testing facilities, medical staff, protective equipment), lowering infection risks, and lessening transport and privacy barriers, that often inhibit in-person testing approaches (9, 13, 14). Unlike self-testing, where individuals check results themselves, self-sampling allows samples to be shipped and analyzed in a laboratory, resulting in longer “time-to-result,” but also higher test accuracies. This approach may also mitigate concerns that self-testing could facilitate underreporting of SARS-CoV-2 infections as self-testers can decide not to report the results (13). In comparison to nasal and (naso-)pharyngeal swab sampling, gargle sampling is often assumed to be more comfortable and has proven to be a reliable tool to detect SARS-CoV-2 (8, 15). However, as the success of testing relies on people's willingness to be tested, it is crucial to assess users' test preferences, test methods' usability, their implementation, and how to best provide potentially needed support (9, 16).

Quantitative studies in the U.S. have found a high (hypothetical) acceptability of home self-sampling with saliva and throat swabs of participants without testing experience (14, 16). However, discrepancies exist between an expressed willingness to use and actual uptake of at-home sampling options (14, 16, 17). Mixed-method studies looking at the post-collection acceptability of sampling in the UK and US among university students and staff or participants, who self-sampled with telehealth guidance, underpinned a high acceptability of self-sampling with saliva and throat swabs without consensus of a preferred method (18, 19). However, these results may show higher acceptances of self-sampling as these studies included telemedicine support and involved specific academic populations. While the latter studies compared swab tests to saliva tests, quantitative studies at schools in Germany and among contact cases or SARS-CoV-2 positive individuals in Canada and India showed that users preferred gargle sampling over saliva, nasopharyngeal or nasal swab tests (20–22).

Qualitative studies in relation to SARS-CoV-2 testing largely focused on barriers and facilitators to testing, the experiences of awaiting and receiving a test result, and implementation experiences in specific study settings such as hospitals, schools, universities and homeless-shelters in Germany, the UK and Denmark (18, 23–28).

While studies have outlined provider perspectives of testing sites, gaps exist about how users experience testing interventions, how test rejectors perceive testing methods and how individuals respond to mail in SARS-CoV-2 tests (29). Studies examined how to improve the implementation of SARS-CoV-2 tests in Germany among specific settings such as homeless shelters and schools (22, 30, 31). This study aims to gain a deeper understanding of how test users and test rejectors perceived SARS-CoV-2 gargle tests and their implementation as mail-in tests with self-sampling and laboratory-based sample analysis. To our knowledge, this is the first study to qualitatively evaluate experiences with SARS-CoV-2 self-sampling among the general population in Germany. The evaluation of both, test takers' and rejectors' perspectives, provides a comprehensive understanding of user preferences. Proceeding from this, we provide evidence and recommendations for healthcare providers, as well as policy and decision makers on how to streamline SARS-CoV-2 gargle and further testing approaches to increase response rates and tests' ease of use.

## Methods

### Study setting

The study took place in southwest Germany in the federal state of Baden-Württemberg, namely Heidelberg town and the surrounding Rhine-Neckar district. In 2020, Heidelberg counted about 158,700 and the Rhein-Neckar district about 548,200 inhabitants (32). Heidelberg has one of the highest life expectancies in Germany and about 70% of its population is of employable age (33, 34). The study region belongs to one of the most prosperous regions in Germany and the economic success is closely linked to an extensive science and research landscape (35, 36). In 2019, Heidelberg had a GDP per capita of 58,209 € and the Rhein-Neckar district of 36,935€ (37).

At the outset of this study in December 2020, (naso)-pharyngeal swab PCR tests were the formally employed test method. Such tests were available at either a high cost or free of charge for a restricted group of people: individuals with COVID-19 symptoms, contact cases, patients or residents prior to admission to health facilities (or facilities with shared housing), as well as staff, visitors and patients/residents of said facilities following a SARS-CoV-2 outbreak (38). Meanwhile, Germany was amidst its second wave that eventually led to the second lock-down on December 16, 2020, and first vaccines against SARS-CoV-2 were authorized by the European Union on December 21, 2020 (39). Over-the-counter self-tests only became available in pharmacies in February 2021, and free RDTs performed by trained staff were introduced in March 2021 (40). In the context of this study, participants were thus confronted with three new aspects: testing for free without meeting test criteria, self-sampling at home, gargling instead of (naso-)pharyngeal swab sampling.

### Study description

**Figure 1:**
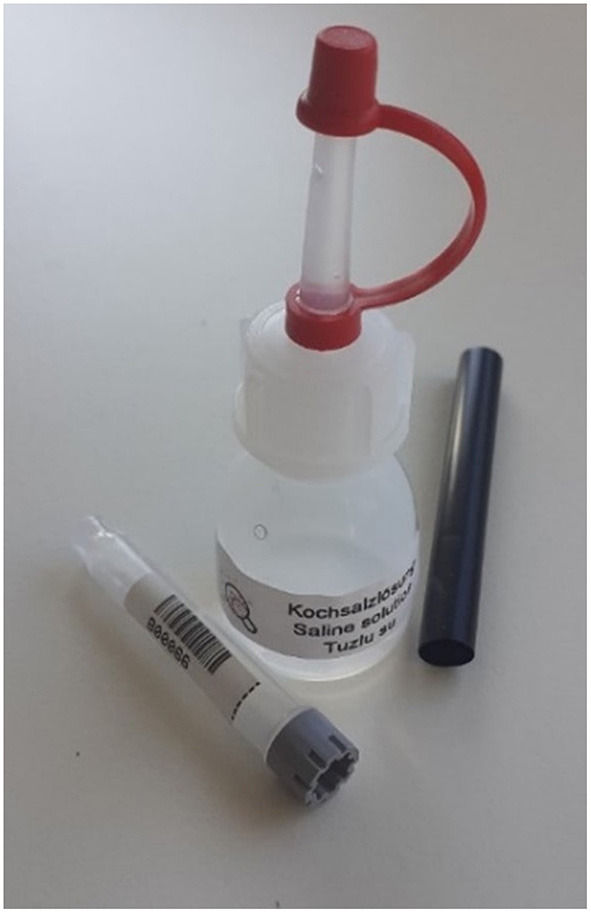
Self-sampling material: Small bottle containing saline solution, straw, test tube.

**Figure 2:**
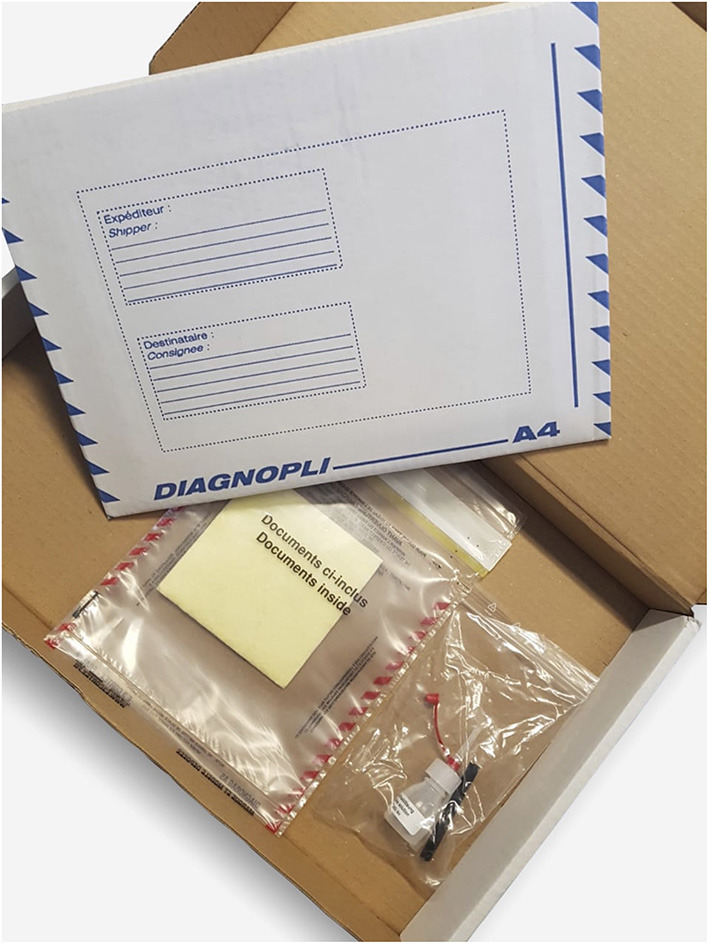
Package received by participants: Shipping carton; protective plastic cover with yellow, liquid-absorbing fleece; plastic bag with testing material.

This qualitative study was embedded in the “CoV-Surv Study,” a two-factorial randomized controlled multi-arm trial with cluster sampling, that evaluated different SARS-CoV-2 surveillance strategies for their acceptability and cost-effectiveness in November and December 2020. Trial details can be found in the study protocol (41). Participants (age ≥7) were selected *via* civil registration services and received either directly a self-sampling kit for themselves or their whole household (arm A) or a pre-screening questionnaire by mail (arm B). If the latter indicated COVID-19 specific symptoms (analyzed by a trained random forest algorithm), they also received self-sampling material by mail. In addition to a photo of the self-sampling material (Figure 1) and the package received by participants (Figure 2), a description of the testing process can be found in Table 1. For the qualitative sub-study, participants were selected from the “CoV-Surv Study” population to share their implementation perspectives of the gargle tests. The results' presentation aligns with COREQ guidelines (Table 2).

**Table 1:** Testing process, study information, and media presence.

**Testing process**
Prerequisite: Sampling in the morning on empty stomach (has proven unnecessary since then); participants were given one sampling kit and asked to test once
1) Gargling with a saline solution (for at least 30 s) 2) Spitting the mouth's content back into the small bottle using a straw 3) Clearing throat and nose through coughing and sniffling (for 30–60 s) 4) Spitting loosened secrete into same small bottle using a straw 5) Dispensing an aliquot into a test tube, using the small bottle's dropping cap 6) Placing the sample with a fleece (as an absorbent material) in a return bag 7) Sending sample to a laboratory *via* mail on the same day 8) Laboratory analyzes sample for SARS CoV-2 using RT-LAMP; in case of a positive test result, same sample is reanalyzed using RT-PCR 9) Participants can check results online from the day after sample arrives at the laboratory and receive help if needed *via* the telephone hotline
**Provided information**
• Cover letters with a website link to further multilingual explanations (in German, English, Russian, Italian, Turkish) and a video showing the self-sampling procedure • Brochure (comprising 58 pages with different segments prepared for various target groups including adults, minors, kids, parents; written and pictorial instructions of the self-sampling, and general study information)
**Media presence**
• The study was publicly referred to in social media (e.g., Twitter, Facebook), local radio and newspapers

**Table 2:** Consolidated criteria for reporting qualitative studies: 32-item checklist (42).

**Item**	**No**.	**Guide questions/description**	**Page**
**Domain 1: Research team and reflexivity personal characteristics**			
Interviewer/facilitator	1.	Which author/s conducted the interview or focus group?	5
Credentials	2.	What were the researcher's credentials?	1
Occupation	3.	What was their occupation at the time of the study?	5
Gender	4.	Was the researcher male or female?	5
Experience and training	5.	What experience or training did the researcher have?	5
**Relationship with participants**			
Relationship established	6.	Was a relationship established prior to study commencement?	No
Participant knowledge of the interviewer	7.	What did the participants know about the researcher?	5
Interviewer characteristics	8.	What characteristics were reported about interviewer/facilitator?	5
**Domain 2: Study design theoretical framework**			
Methodological orientation and theory	9.	What methodological orientation was stated to underpin the study?	4–6
**Participant selection**			
Sampling	10.	How were participants selected?	4–5
Method of approach	11.	How were participants approached?	4–5
Sample size	12.	How many participants were in the study?	4–6
Non-participation	13.	How many people refused to participate or dropped out? Why?	4–5
**Setting**			
Setting of data collection	14.	Where was the data collected?	5
Presence of non-participants	15.	Was anyone else present besides participants and researchers?	5
Description of sample	16.	What are the important characteristics of the sample?	6, 22
**Data collection**			
Interview guide	17.	Were questions provided by authors? Was it pilot tested?	5
Repeat interviews	18.	Were repeat interviews carried out? If yes, how many?	No
Audio/visual recording	19.	Did the research use audio or visual recording to collect data?	5–6
Field notes	20.	Were field notes made during and/or after the interview?	5–6
Duration	21.	What was the duration of the interviews or focus group?	5–6
Data saturation	22.	Was data saturation discussed?	5
Transcripts returned	23	Were transcripts returned to participants for correction?	No
**Domain 3: Analysis and findings data analysis**			
Number of data coders	24.	How many data coders coded the data?	5–6
Description of coding tree	25.	Did authors provide a description of the coding tree?	No
Derivation of themes	26.	Were themes identified in advance or derived from the data?	5–6
Software	27.	What software was used to manage the data?	5–6
Participant checking	28.	Did participants provide feedback on the findings?	
**Reporting**			
Quotations presented	29.	Were participant quotations presented to illustrate the themes/findings? Was each quotation identified?	Yes, 5–11
Data and findings consistent	30.	Was there consistency between the data presented and findings?	Yes, 5–11
Clarity of major themes	31.	Were major themes clearly presented in the findings?	Yes, 5–11
Clarity of minor themes	32.	Is there a description of diverse cases or minor themes?	Yes, 5–11

### Theoretical underpinnings

We drew from aspects of the “Consolidated Framework for Implementation Research” (CFIR) to gain an in-depth understanding of the mechanisms impacting a tests' implementation and to formulate recommendations for future implementers (43). The CFIR comprises five major domains to guide formative evaluations of interventions' implementation (implementation process, characteristics of individuals involved, intervention characteristics, outer and inner setting). We emphasized CFIR components that are relevant to user experiences (excluding components at provider and organizational levels as this was not the focus of our study).

### Sampling procedure

We purposively sampled participants to maximize variation of ages, sex, educational backgrounds, study arms and test/questionnaire up-take or rejection. We included test rejectors' perspectives as they still experienced the tests' implementation and could provide insight into their impressions of the sampling method. Recruitment started on December 16, 2020, a month after self-sampling kits were first mailed. We contacted test up-takers *via* mail and e-mail, and test rejectors exclusively *via* mail due to limited available contact information. After no test rejector accepted the interview invitation following the first letters, we changed the procedure, searching instead for online listed telephone numbers and calling participants. The response rate was higher among test takers (~43%) than test rejectors (~4%). Reasons for interview rejection were inability to participate (language barrier, health reasons, death, no memory of study), study-related factors (distrust in data security, online interview) and disinterest. Ultimately, 67 individuals (37 takers; 29 rejectors) agreed to be interviewed and recruitment stopped on February 12, 2021, after data saturation was reached within each respondent group.

### Data generation

Interviews were conducted using a semi-structured in-depth interview guide that we pretested with individuals external to the study (*n* = 19) of different ages, education, and professions. The interview guide covered reasons for or against self-sampling; perception of the gargle test before and, if applicable, after sampling; perception of the implementation and suggestions for improvement; and implications of test results, if applicable. The slightly different interview guides for test takers, rejectors and positively tested are attached as Supplementary Data 1. Depending on interviewee's choice, the 67 interviews were conducted by phone or video calls in German, English or French. Although participants were asked to be alone, twice participants' partners or legal guardians were present. Rarely, interviews were interrupted due to bad internet connection or empty phone batteries, but all interviews were completed. We summarized essential aspects of the interview at the end to allow for feedback or clarification from the interviewees.

### Reflexivity

FU (cis-male) and FR (cis-female) conducted the interviews as their first research project. Both are studying medicine and experienced the pandemic in Germany. They had undertaken training in qualitative research and interviewing skills before and during data generation. Their professional background and solidarity-based approach to the pandemic led them to welcome testing as a measure against the pandemic.

### Data analysis

FR or FU interviewed all participants once. Interviews were audio recorded and interviewers took field notes during and after interviews. Interviews took on average 1 h (shortest 26 min; longest 110 min). The research team, including senior authors, debriefed regularly after interviews (44). One interview was excluded from analysis because the audio recording failed, and the study group was thus reduced to 66 participants. Audio recordings were transcribed verbatim, with 30 recordings pre-transcribed using “f4transkript” software and manually corrected afterwards, while the remaining audio recordings were transcribed entirely manually (45). Impressions of the test method from participants' acquaintances or relatives mentioned during the interviews were included in the analysis. We used investigator triangulation with two researchers generating and analyzing data (coded the same interviews at the beginning to validate and finalize the codebook and constant discussion during the coding), in close collaboration with senior researchers *via* regular debriefings after and in between interviews. Analytical categories were derived inductively from the data drawing from principles of grounded theory (46). The data were analyzed sequentially during and after data collection using thematic analysis (47). Closely accompanied by senior authors, FR and FU created a codebook with codes that derived during data collection, debriefings, and analysis of first, especially rich transcripts. FR and FU continued coding together using “NVivo 12 Pro” (RRID:SCR_014802) and later separately while regularly consulting and mutually checking for (dis-)agreement. No member checking was done. Once we had identified an emerging phenomenon, we looked for disconfirming cases and data that could disprove a theory. We identified the following implementation related themes: first reaction of recipients, trust in the study, self-efficacy, communication (provided information, reminders, support system, and test result), perception of the self-sampling method and its diagnostic accuracy, and timing of the study. To present the results in a way that is particularly valuable for future test implementations, we arranged identified themes according to appropriate CFIR components: “intervention process” (divided into “intervention engaging” and “intervention execution”), “characteristics of individuals involved,” “intervention characteristics” and “outer setting.” Where deemed necessary, subdomains were added to the CIFR components such as “first reaction of recipients” and “reminders.”

## Results

The study group consisted of 66 participants with the following characteristics: 37 (56.1% of all participants) test takers and 29 (43.9% of all participants) test rejectors, 31 (83.8% of test takers) negative and 6 (16.2% of test takers) positive test results, 36 (54.5% of all participants) women and 30 (45.5% of all participants) men of different age groups and school leaving qualifications (Table 3).

**Table 3:** Characteristics of participants.

**Sample characteristics**	***n*** **(%)**
**Decision to test**
Test uptake	37 (56.1%)
Test rejection	29 (43.9%)
**Sex**
Female	36 (54.5%)
Male	30 (45.5%)
**Age**
<18	1 (1.5%)
18–29	14 (21.2%)
30–44	10 (15.2%)
45–59	21 (31.8%)
60–80	18 (27.3%)
>80	2 (3.0%)
**School leaving qualification**
No school leaving qualification	1 (1.5%)
Low education level (9 years of schooling)	11 (16.7%)
Middle education level (10 years of schooling)	17 (25.8%)
High education level (11–13 years of schooling)	31 (47%)
Missing	6 (9.1%)
**Test result**
Positive	6 (16.2% of test takers)
Negative	31 (83.8% of test takers)
**Job classification** ^a^
1) Managers
Production and specialized services managers	2 (3%)
2) Professionals
Science and engineering professionals	1 (1.5%)
Health professionals	4 (6.1%)
Teaching professionals	11 (16.7%)
Business and administration professionals	6 (9.1%)
Information and communication technology professionals	1 (1.5%)
Legal, social and cultural professionals	1 (1.5%)
3) Technicians and associate professionals	6 (9.1%)
4) Clerical support workers	3 (4.6%)
5) Services and sales workers	7 (10.6%)
6) Skilled agricultural, forestry and fishery workers	2 (3%)
7) Craft and related trades workers	8 (12.1%)
8) Plant and machine operators and assemblers	1 (1.5%)
Students/pupil	8 (12.1%)
Missing	5 (7.6%)

a According to “International Standard Classification of Occupations 2008” (48).

### Implementation process—intervention engaging

#### First reaction of recipients

The first reaction of both, test takers and rejectors, upon receiving test material was often surprise, followed by responses that ranged from delight (“*like Christmas presents”* [male, 22, uptake]) to confusion (“*Why me of all people?”* [female, 65, rejection]) to senselessness (“This is nonsense” [female, 62, rejection]). Respondents, including test rejectors, generally expressed gratitude because the tests enabled knowing one's status at a time with very limited access to tests and, more broadly, the study enabled to support broader efforts to address the pandemic at no personal financial cost. However, tests were also perceived as burdensome as they came at a busy time of the year (around Christmas) and at a time when pandemic fatigue was propagating. Rarely, respondents wrongly associated the study with SARS-CoV-2 vaccinations and rejected the study without knowing what it entailed.

Both test takers and rejectors intuitively trusted the study because they were contacted by letter instead of by phone; the university of Heidelberg organized the study and material showed official logos. “*I found it very, very reliable. Which I think is just super important in the beginning when you get that.”* [male, 23, uptake]. Furthermore, participants verified information online and understood that the municipality provided personal data. However, misinterpretations arose too, and some respondents believed they were contacted due to previous hospital stays, participation in other studies, former or up-comping SARS-CoV-2 tests or acquaintances in quarantine. “*But I've only just been tested, I'm negative.”* [male, 60, rejection]. For a few respondents, the study invitation triggered a fear of being infected or “*Oh, God, I thought the notification was coming, that we have to go into quarantine.”* [female, 24, uptake].

Some respondents explained that being contacted without prior notification, triggered distrust. Moreover, distrust was fueled by not understanding how personal data was accessed or fear of data fraud and of analyses of saliva samples for other purposes. “*I don't know what kind of shenanigans they might be up to.”* [male, 46, rejection]. Distrust was further increased by more individual factors such as being uninformed about test capabilities or being “*always a bit anxious”* [male, 83, uptake].

#### Perception of provided information

Across sexes and ages, test takers and rejectors stated the brochure contained “*good explanations”* [female, 24, uptake], and facilitated to understand subject-specific vocabulary. Participants appreciated that the brochure contained wording for several target groups, including simplified summaries. However, the amount of information was considered too much by both test takers and rejectors. “At first, I saw the 60 [brochure's pages] and was shocked!” [male, 62, uptake]. Participants estimated “*[…] this takes a while […] to read that.”* [male, 60, rejection]. Participants appreciated pictures depicting the sampling steps to better understand the sampling procedure. A video on the website facilitated sampling and increased self-efficacy. Furthermore, participants emphasized the importance of multilingual online options.

Despite provided information, misunderstandings arose about the study duration and the consequences of participation. “*And then I thought, three weeks,”* one participant [female, 46, rejection] said, in reference to the frequency of having to test for the study (limited to a single test) “*I'm not going to be able to do that.”* [female, 46, rejection]. Participants criticized a lack of information regarding when samples must be returned and whether testing could be postponed to a more convenient time. “*[...] if I get the test kit today, I should preferably send it off tomorrow. I didn't find that anywhere, otherwise I would have hurried a bit.”* [female, 63, uptake]. Often information on the study's timeframe was only received *via* media or reminders. Additionally, participants asked for more information about the saline solution (for example storage life, implications of swallowing). At least a few male test takers and rejectors described feeling “*a bit scared”* [male, 24, rejection] because the sample packaging included symbols indicating biological substances, a requirement to send samples of bodily fluids *via* mail. A biohazard symbol raised concerns about the saline solution's potential harmfulness in case of swallowing and compelled questions such as “*[...] how dangerous is it to put that then in the waste?”* [male, 23, uptake].

### Implementation process—intervention execution

#### Support system—hotline

Participants deemed the possibility to call a hotline during the intervention reassuring and helpful as a contact option for respondents without internet, to receive their test result, correct mistakes or clarify questions about the sampling.

#### Reminders

Reminder letters surprised and prompted many respondents to test. “*When the second letter came, I said: I'll do it, then […] I'm not guilty then for it [the study] not working.”* [male, 62, uptake]. Others felt pressured, or questioned why a letter would come almost immediately after the test arrived. “*I was incredibly annoyed that a letter came two days later”* [female, 55, rejection]. Participants described sensing that communication within the study team was “*weird”* [female, 56, uptake], noting that reminders arrived after samples had been submitted or after respondents had confirmed their study participation *via* the hotline. For respondents who had already submitted samples, the reminder letter triggered uncertainty about a possible loss of samples in the mail. Moreover, respondents criticized the letter's use of “*pressurizing*” [male, 62, uptake] wording, which called into question the voluntary nature of study participation.

#### Characteristics of individuals involved

How confident respondents felt to self-sample was closely intertwined with their perception of the test method and the information provided. Overall, respondents who took up the test intuitively felt capable to sample or were reinforced in their ability to perform the test once they: trusted the study team to sort out samples that were sampled incorrectly by participants, watched others test first, were professionally trained to develop, or use similar tests, and could draw from testing experience. Others felt unable to sample correctly because they felt incompetent with tasks deemed medically complex; wanted more personalized instructions; feared making mistakes that could jeopardize a study; or were physically unable to perform self-sampling due to advanced age, injured oral mucosa or an “*extreme gag reflex”* [female, 56, uptake]. Furthermore, participants worried self-sampling may not be feasible for individuals with disabilities or young children. However, participants whose children (age ≥7) self-sampled perceived the method as child friendly.

Moreover, participants with a scientific background or who generally supported measures against the pandemic tended to be more appreciative of the test method. In contrast, participants distrusting measures expressed “*This is all exaggerated! […] I don't think much of [tests] myself* ” [male, 79, rejection] and questioned the general meaning of testing.

### Perception of intervention characteristics

#### Relative advantage

At first glance, test takers and rejectors were surprised by the test method and its perceived simplicity. “*I wasn't aware that there was such a possibility to do such a test and I was amazed and eager to see how it works*.” [female, 32, uptake]. Partly, test rejectors were irritated and wondered “*where the swab was. […] I thought, well, they probably forgot it*.” [female, 65, rejection]. While some test takers were relieved to sample without swabs, other test takers and rejectors imagined the test to be too complicated or unfeasible. Few test takers feared to be unable to gargle *per se* or for a long duration.

After self-sampling, test takers generally found the test “*really easy to carry out*” [female, 32, uptake] and “*uncomplicated*” [female, 72, uptake] and participants, who had initially deemed the test too complex, often changed their minds. However, some test takers struggled especially with gargling which they found “*strenuous*” [female, 56, uptake], the dispensing of an aliquot or clearing secretions from throat and nose which they considered “*disgusting*” [male, 55, uptake] and difficult causing insecurity about correct test execution. “*[…] To, uh, kind of bring the inside of the nose into the throat and get that out through the mouth. I didn't manage that, [...] I really tried hard [...]*.” [female, 43, uptake] Although only few respondents considered the saline solution disgusting and reported an aftertaste lasting for hours, for those affected this became a decisive factor in the choice against the gargling test as a preferred method.

In comparison to (naso-)pharyngeal swab tests, many test takers and rejectors across sexes and ages appreciated gargling as being more “*pleasant*” [female, 20, uptake] and “*MUCH more comfortable*” [female, 20, uptake]. “*This swab in the nose, [...] the idea alone is not so good*.” [female, 32, uptake] Still others deemed the gargling as unpleasant or more complicated than swab tests, preferring “*[…] a swab in the throat, move it around for 30 seconds [...] um, that would make it [...] easier […]*.” [male, 30, rejection]. Most test takers felt more confident to self-sample correctly by gargling than using a (naso-)pharyngeal swab test: “*I don't think I would have the courage to ram it so far into my brain [...]*.” [female, 57, uptake]. “*I just think that ordinary people [...] are not thorough enough. That's why I thought the idea of the spit test was a brilliant one, because you can't do too much wrong*.” [female, 58, uptake].

Mostly younger test takers appreciated the aspect of self-sampling at home as less time-consuming or strenuous, more flexible than testing on the spot. Respondents emphasized particularly the “*advantage of not being at risk of infection*” [male, 29, uptake]. Furthermore, self-sampling was hoped to “*relieve the burden on the health system*” [female, 33, uptake] by requiring fewer resources. Both test rejectors and up-takers, especially over 45 also saw disadvantages of self-sampling such as higher test complexity, perceived higher skill and time requirements, having to trigger discomfort oneself and higher uncertainty about correct test execution. “*[…] I would prefer to […] go somewhere. I don't have to read brochures. I don't have to be uncertain*.” [male, 58, uptake]. Participants worried because they only had one attempt to sample correctly, as they only had one bottle of saline solution. While a laboratory-based analysis was generally appreciated, some respondents wished for “*a test where you could evaluate yourself at home*.” [female, 44, rejection] to receive faster results with less effort. While many trusted trained staff more than themselves to sample correctly, a few trusted their test result mainly due to self-sampling.

#### Time of sampling

Sampling in the morning, on an empty stomach was considered “*making [testing] difficult*” [female, 18, rejection] and “*disgusting*” [female, 58, uptake]. Participants felt that they did not have enough saliva, or they delayed sampling due stressful mornings, struggles to change morning routines, accidentally brushing teeth, eating or drinking.

#### Packaging and shipping

While some respondents appreciated the overall layout, others criticized the test's packaging because it contained “*a bunch of plastic*” [male, 29, uptake], raising environmental concerns. Test-rejectors demanded the option to opt out before receiving material to reduce waste. The inclusion of plastic straws, which had been banned half a year prior in Germany, sparked questions such as “*Shouldn't these not even exist anymore?*” [female, 42, uptake].

Some respondents highlighted the good manageability and preparation of postage-paid, pre-addressed envelopes. Families that were sent several tests to facilitate pooled testing, described wanting several return envelopes to return individual samples immediately. At times, participants wondered how to best protect samples for shipping and improvised covers because they did not know how to use the enclosed protective covering. While some respondents praised shipping *via* mail as convenient, others worried tests could get lost, or they found being asked to go to a post box or office stressful (of note: outgoing postage in Germany is not usually sent from residential addresses).

#### Communication of test results

Generally, receiving test results online was considered fast and convenient. While participants noted that relying on the internet inhibited engagement from those who lack connectivity, test takers who lack connectivity (typically older participants) described the ease of calling the hotline. Test takers described unclear communication regarding how to retrieve test results; and having waited for results to arrive, not knowing that they had to check results themselves. Participants, that took the test, wished for active feedback on test results or at least a notification that results could be checked online. Others found it unnecessary to retrieve results, as they assumed a positive result would entail outreach from the study team or health authorities.

Some found the waiting time for results short, while other test takers, especially those who (voluntarily) quarantined themselves, found it too long, causing discomfort and anger. “*It took ages to get the results. I was a bit annoyed […] and then I didn't look at all anymore*.” [female, 72, uptake]. Based on other health-care experiences, test takers usually considered longer waiting periods for test results as an indication of a negative test result, while to a lesser extent, participants described being more attentive regarding potential symptoms as they increasingly feared a positive result. Test takers and rejectors highlighted that receiving results several days after sampling undermined the test's purpose because contact tracing became less feasible, and infection could have occurred in the interim. Participants described a desire to include test results in the official SARS-CoV-2 contact tracing app used in Germany.

#### Evidence strength and quality—test accuracy

Test takers described trusting the tests' accuracy because results met personal expectations “*[...] feel fine. Then it*'*s [test result] right.”* [female, 63, uptake] and participants trusted broader study aspects (type of test, option for follow-up PCR testing of same sample, study's institutional affiliation).

Although participants who tested positive trusted their test result overall, doubts arose because participants did not understand the double analysis process of the same sample *via* RT-LAMP and RT-PCR and expected to receive a confirmatory PCR test that included renewed sampling. Few test rejectors distrusted gargle liquid tests due to an alleged generally low accuracy or believing that viral loads are lower in saliva. A few test rejectors generally distrusted test accuracies, among other things due to media reports about poor test qualities such as that rapid diagnostic tests were false positive through the addition of soft drinks. “*I can't believe it [quality of tests] anymore. [...] They dribbled a little Coca-Cola onto a test strip and then it was positive!”* [male, 70, rejection] Comparing our study test to other testing methods, participants generally believed “*rapid diagnostic test is less reliable anyway.”* [male, 71, uptake], while swab tests with PCR evaluation were often described as a benchmark for accurate tests and considered to have comparable or higher diagnostic accuracy. “*If I'm honest, I think only PCR tests are accurate.”* [female, 44, rejection].

### Outer setting—timing of the study

Participants described how receiving tests in the mail around the holiday season sparked conflicting emotions: on one hand, a negative result could facilitate participation in social events, on the other hand, a positive result would hinder important events or awaited reunions. Among other things, this led to testing being postponed to convenient times such as right before social events without participants realizing that the samples may arrive too late for the study and could thus not be analyzed anymore.

## Discussion

This study uniquely demonstrates in-depth how test takers and rejectors perceived mail-in SARS-CoV-2 gargle tests and their implementation as an active surveillance strategy. The identified implementation recommendations further apply to other SARS-CoV-2 tests and testing strategies such as diagnostic and screening testing. While the perception of gargle sampling ranged individually from disgusting and overly complicated to simple and brilliant, the method was generally well-accepted and appreciated as a more pleasant alternative to (naso-)pharyngeal swabs. Communication (first contact; quantity and content of information; reminders; support system; timeframe of when to return samples, receive results and study duration) and trust (in study and test method) served as key factors influencing the intervention's acceptability. While the amount of information and perceived test complexity initially overwhelmed many, illustrations and a video of test steps were helpful to complete sampling.

Participants considered self-sampling convenient and important to reduce infection risks during testing, but at times more effortful and causing uncertainty about correct test performances. Although participants mostly of higher age felt overchallenged by self-sampling, self-efficacy was high for many respondents and accurate self-sampling was found more feasible with gargling than swab tests.

Consistent with studies and commentaries assuming saliva and gargling sample tests are as or more accepted than (naso-)pharyngeal swabs, our results show that gargle sampling was generally positively perceived (18, 20–22). Furthermore, our results show that the perception of the gargle test varied widely, complementing findings of a quantitative study in Canada in which individual test preferences caused a variability of discomfort levels of both, (naso-) pharyngeal swabs and saliva tests, that ranged from minimal to extreme (49). Consistent with findings of Granger et al. (50), evaluating saliva sampling, which resembles gargling sampling, inter-individual differences in sampling abilities underscored that gargle sampling can be difficult for old individuals or depending on gag reflex, disease states and cognitive status. After testing participants often considered the method less complicated than anticipated, which underscores that test acceptability can change considerably after a testing experience (51). To our knowledge respondents' impression to feel more confident to self-sample correctly with gargling than (naso-)pharyngeal swab testing has not been shown in other studies.

The fact that self-sampling at home was perceived as beneficial complements findings of a US survey that more people are willing to self-test at home, which includes self-sampling, than be tested elsewhere (14). The appreciation of lower infection risks with home self-sampling is consistent with qualitative findings on SARS-CoV-2 testing showing increased fear of infection risk at testing facilities (28). However, to our knowledge, no other studies have shown that some participants found self-sampling more strenuous than being tested at a testing site.

Although gargle self-sampling itself increased participants' self-efficacy and some participants trusted the test results mainly due to having self-sampled, our results emphasize the need to empower testing confidence, especially as success of self-sampling depends on users' belief to be able to self-sample. In contrast, quantitative and mixed-method studies in the US and UK found a more homogenous picture of high self-efficacy and self-sampling feasibilities of saliva tests (18, 19). However, these studies may show higher self-efficacy as participants only sampled saliva, received support by telemedicine or belonged to specific study groups (university staff, students) (18, 19). To increase self-efficacy, Conserve et al. suggest using strategies proven successful in HIV self-testing such as online, real-time instructions (9).

In contrast to results of a mixed-methods study at a UK university, which identified no significant concerns about saliva tests (without gargling), our participants expressed distrust of a sample analysis for other purposes and a potential harmfulness of the saline solution (18). This discrepancy may be explained by differing study populations (university setting vs. general population) or by the fact that in our study, participants were contacted at home without prior notice and had to gargle with an additional liquid solution.

The negative impact of too much information (deterring and less information being absorbed) is consistent with qualitative findings on unspecified swab and saliva sample testing in a university setting in the UK (18). Studies about bowel cancer screening kits also highlighted the negative impact of complicated instructions and a mixed-method study about SARS-CoV-2 testing demonstrated the need for detailed and clear diagrams, especially for a method similar to gargling, saliva sampling, because it was most frequently described as unfamiliar and complex (19, 52). In the context of our study, extensive information material was requested by the ethics committee and may be reduced or made available online and *via* various information channels in routine SARS-CoV-2 surveillance systems. We further suggest informing users about the key aspects of correct sampling, such as that the aim of gargling is to collect virus-containing mucus cells from the throat regardless of the amount of saliva, allowing to gargle with a dry mouth. Reminders have proven effective in increasing immunization rates and should be implemented as a valuable tool (53). However, our results indicate that attention must be paid to appropriate timing and polite wording of such reminders. Regarding the communication of test results, longer waiting time reassured some to be negative, but also caused anxiety in alignment with study findings indicating that awaiting SARS-CoV-2 test results triggers anxiety of positive results (18). Waiting times in this study were mainly caused by postal delivery delays, which high-lights how mail, as a potentially more convenient delivery method, depends on external factors such as the postal volume in a pre-Christmas period. Postal delivery delays may, for example, be avoided by community collection points to drop off samples. Participants wished for timely results and preferred having results delivered (electronically or by phone) rather than undertaking a search for results.

While gargling has been promoted as the least invasive sampling method, it has hardly been implemented at large scale. Projects that we know of are for instance: “Alles gurgelt” in Vienna, Austria, that uses a screening testing strategy aiming at testing many individuals with gargle test kits available for free at supermarkets, sample drop-off at collection points and PCR results available within 24 h; and the study “WICOVIR” that uses at-home gargle testing and pool PCR testing in German schools (30, 54). While we are not aware of any published research on the implementation and user experience of “Alles gurgelt,” Kheiroddin et al. (30) have evaluated and published how to efficiently implement SARS-CoV-2 gargle-based pool PCR testing, but focus on schools and not the general population. Our findings may inform the implementation of according testing approaches and further active surveillance strategies, while at the same time evaluations of mentioned projects may reveal complementary insights.

### Limitations

This study provides in-depth qualitative data about the implementation experiences of both test users and rejectors of SARS-CoV-2 gargle tests. The study population consists of participants with varying characteristics, allowing insight into diverse perspectives, in a rural and urban study setting. However, this study also has limitations. Due to the rapidly changing nature of the pandemic, referring policies and increasing testing opportunities, opinions on SARS-CoV-2 testing may have changed in the meantime. However, lessons learned from this study are still useful to understand the communities view on testing and improve testing strategies and the implementation of new interventions. Although we purposively sampled to maximize diversity among participants, sampling may have been biased as participants appreciating the study aim and gargle sampling may have been more willing to be interviewed. We balanced this potential bias by also sampling test rejectors. Since the study took place in a region of Germany with high socio-economic status, we sampled people with different socio-economic status to avoid the bias of only talking to highly educated and high earners. Courtesy and social desirability bias may affect our data if participants did not express dissatisfaction or gave responses perceived to satisfy interviewer. We tried to minimize these biases by building rapport, probing and reflexivity.

## Conclusion

Our findings suggest that SARS-CoV-2 active surveillance strategies should integrate in advance of an intervention implementation: large-scale information campaigns; diverse information and communication channels (e.g., radio, newspapers, health authorities); support systems for participants (e.g., hotlines, online contact forms); official support from policy makers and health authorities. Communication and trust are key elements to focus on while implementing (new) testing interventions. Provided information on testing strategies and test steps must be minimized in its quantity, while conveying essential aspects including the rationale of testing and time frames and should include visualizations such as videos of sampling and retrieval of results. A summary of key recommendations for implementing SARS-CoV-2 tests can be found in Table 4. Mail-in gargle self-sampling proved successful in our study. The method was perceived as pleasant and convenient and increased individuals' confidence to correctly sample, while enabling high test accuracies through a laboratory-based analysis. However, it is important to consider user limitations of gargle self-sampling and to offer a range of possibilities to get tested to adapt to the needs and preferences of users. Considering diverse user opinions found in this study, further exploration is needed regarding users' sampling preferences. Given the lack of data involving SARS-CoV-2 test rejectors, we encourage researchers to include this perspective as well to effectively improve testing interventions. Further research is needed of how to increase self-efficacy and trust in testing methods.

**Table 4:** Key recommendations for implementing SARS-CoV-2 tests.

**Stages**	**Recommendations**
**Intervention engaging**
Communication	First contact: Establish trust by contacting people with prior notice and media presence Access to information: - Provide large-scale promotion and information campaigns about the testing strategy/project *via* diverse information channels - Arrange information on sampling steps with depictions visible at first glance and provide a webpage with video instructions on self-sampling (in languages commonly spoken in a region of interest) - Integrate general information about: □ how personal data was obtained □ how data security is ensured □ timeframe of testing project; time (limit) to return samples □ in case of a new test method: emphasis of method's novelty and difference to established methods □ information about kit components: composition, risks, preservability □ key aspects for correct sampling □ how sample is analyzed; what sample components are analyzed Limit use of unknown signs (e.g., biohazard symbol) Reminders: Implement reminders. Pay attention to polite phrasing and timing Support system: Offer personal contact (phone hotline, online contact form, telehealth session for first sampling). Ensure link between support system and executing project parts to respond to peoples' concerns Test result: Ensure timely, online accessible results. Inform about availability of results
**Intervention itself**
Relative advantage	Test characteristics: Consider the characteristics of available test methods including tests' complexity, discomfort, feasibility, and user preferences. Consider addressing inter-individual differences in method preferences by offering a choice of multiple test methods Test execution: Adapt choice of test method to age, physical and cognitive abilities of a target group. Consider benefits of self-sampling (reduced infection risk, less travel and time spent, lower costs for health system, high test accuracy due to laboratory-based analysis) vs. challenges (self-efficacy) before deciding on a method
Design quality	Minimize test steps, material, and limitations (e.g., testing on empty stomach/in the morning). Take environmental aspects into account when choosing kit material
Packaging/shipment	Avoid unnecessary material. Provide packaging with clear instructions for use. Consider dependencies of shipment methods on external factors. If tests are sent *via* mail: provide updates on samples' shipping status and consider potential delivery delays (e.g., in a pre-Christmas period)
Evidence strength	Provide clear information about test method's accuracy compared to other methods. Consider participants with a positive SARS-CoV-2 test value further confirmation of result with reference methods
**Outer setting**
Political/epidemiological context	Official support from policy makers and health authorities is key Assess local need of self-sampling and testing for SARS-CoV-2 before implementation Define passive or active surveillance system Adapt interventions to the epidemiological context and local policies Counteract that receiving mail-in tests during high incidences period may trigger fear of quarantine and being infected by a clear communication Consider that users expect test methods commonly used at the time of an intervention. If applicable, highlight the novelty/positive aspects of an intervention
Socio-cultural factors	Include sociocultural factors (holidays, religious celebrations, etc.) in the timing and communication of an intervention

## Data availability statement

The generated datasets will be made available by the authors upon agreement from the data owners and after a period of 12 months after the publication of the paper. Requests to access the datasets should be directed to freda.roehr@uni-heidelberg.de.

## Ethics statement

The study involving human participants was reviewed and approved by the Ethics Committee of the Medical Faculty of the University of Heidelberg (S-790/2020). All study participants (or their legal guardians) gave informed, verbal consent that was recorded before interviews. Verbal consent and interviewee's personal data were audio recorded separately to allow pseudonymization of results. Personal identifiers such as names of people and places were removed from transcripts. Interviewees could stop the interview at any time.

## Author contributions

FR: formal analysis, investigation, data curation, interpretation of data, writing—original draft, writing—review and editing, visualization, and project administration. FU: formal analysis, investigation, data curation, writing—original draft, review and editing, and administrative support. AD: conceptualization and design, methodology, writing—review and editing, project administration, and supervision. SA: software, implementation of data logistics, resources, development of sampling procedure and mailing logistics, writing—review and editing, and obtaining funding. RB: resources, set up and led the laboratory, development of sampling procedure and mailing logistics, and writing— review and editing. MK: resources, set up and led the laboratory, development of sampling procedure and mailing logistics, writing—review and editing, and obtaining funding. LB: resources, development of sampling procedure and mailing logistics, and writing—review and editing. TB: conceptualization, methodology, supervision, review and editing, and obtaining funding. AJ: conceptualization qualitative component, methodology, writing—review and editing, and supervision. SM and AS: conceptualization qualitative component, methodology, formal analysis, data curation, writing—original draft, writing—review and editing, visualization, supervision, and project administration. All authors contributed to the article and approved the submitted version.
