# Supplementary material for: From disgusting and complicated to simple and brilliant: Implementation perspectives and lessons learned from users and rejectors of mail-in SARS-CoV-2 gargle tests
Source: Front Public Health. 2023 Jan 5;10:1024525. doi: 10.3389/fpubh.2022.1024525 (PMC9850099; doi:10.3389/fpubh.2022.1024525)
Supplement: Supplementary file 1 [file Data_Sheet_1.doc]

Supplementary Material

# Supplementary Data 1: In-depth Interview Guides

**1.1 In-depth Interview Guide for Test Rejectors and Test Takers Who Received a Negative Test Result**

Start for test takers and rejectors:

- How are you dealing with the Coronavirus during your daily life? / If I mention the Coronavirus, what comes to your mind?
- In your view, how has the pandemic/Coronavirus affected you/people around
  you (in your daily life, personal experiences)?
- How high do you estimate your risk to get infected with the Coronavirus?
- What do you think about the severity of COVID-19 (own risk, risk for people in the household)?
- What are your information resources about COVID-19 and the pandemic in general?
- What do you think about the testing situation in your area (access to tests, relevance of testing)?
- In your view, what role does testing play in the pandemic?
- How accessible do you deem tests?
   What are things that make the access difficult (for you personally or in
   general)?
   What facilitators are there to get tested (for you personally or in general)?

Follow-up part for individuals who took the test and received a negative test result:

- Have you already heard of the gargle test before this study?
- How was your experience with the gargle sampling? Please guide me through the sampling experience.
- How comfortable did you feel doing a test yourself?
- What do you think about the testing procedure?
  Was there anything that made it hard to do this test?
- How did you decide to take the test? What did you weigh for or against taking the test?
- What were your expectations regarding the test? What consequences did you think the test would have on your life before conducting the test? How did you feel before you took the test?
- Our records indicate that you received a negative test result. Is that true?
- What was your immediate reaction after receiving the test result?
- What were your feelings towards the test result?
- Did you change something in your life before receiving the test result?
- Did you change something in your life after having received the test result?
- How would you recommend improving the gargle test and its implementation?
- How would you recommend improving: the information available; the act of doing the gargle test; the shipment of the test; communication of test results?
- What should remain the same?

Follow-up part for individuals who rejected the test:

- We sent out the test-kits to thousands of people. Thousands of people responded and sent us their tests back, thousands of people did not respond (and presumably did not take the test). This includes many people, and we would like to understand better why people might not like to do this test. Can you imagine why people would not like to take the test?
- Our records indicate that we did not receive a response and we were unable to include data from your household within the study. / Our records show that we did not receive a sample from your household. Can you confirm that you declined to participate?
- If ‘no’: Would you elaborate on that?
- If ‘yes’: How did you decide to not take the test?
   What is your opinion about the gargle test?
   How trust-worthy do/did you think the test result is/was? (In comparison to
   commonly used tests? ((naso-)pharyngeal swab tests with PCR evaluation)
- If we would like to redo this study/implement the gargle test in the future, what do you think could be changed to encourage citizens to participate in an intervention like this?

Follow-up part for test takers and rejectors:

- Have you ever thought about getting tested before? If yes, did you get tested before?
- What kind of test was it?
  If applicable, how would you compare the former test you have done and the gargle test? If applicable, how was your experience with that test?
- Was there a difference in the amount of time spent for the testing/sampling?
- How did you decide to [not] get tested? What influenced your decision?
- If, applicable, would you like to tell us whether the result was positive or negative?
  How did the test result influence you back then? Did you change your behaviour after getting tested?
- If you could choose one test method to test with in the future, which one would you choose?
- Do you trust one test method more than another?
- Would you be willing to get tested in the future?
- In what situation would you be willing to get tested?
- What consequences would regular testing have on your life? Specifically in your role as [mother/father/employee/employer]? Would regular testing have any personal advantages or disadvantages?
- What advantages/disadvantages would regular testing have for society?
- Did the gargle test/this study ultimately have an affect/change on how you see testing/the diagnosis Covid-19?
- How did your opinion about COVID-19, SARS-CoV-2 tests, and quarantine change during the pandemic?
- Is there anything I did not ask you, that I should have asked you?
- Would you like to add anything else?
- Do you have any questions that you would like to ask me?
- How was your experience with conducting the interview?
- What would you have liked to be different? Would you have preferred a different way to conduct the interview?
- Did you experience any technical difficulties?

**1.2 In-depth Interview Guide for Test Takers Who Received a Positive Test Result**

- How are you dealing with the Coronavirus during your daily life? / If I mention the Coronavirus, what comes to your mind?
- In your view, how has the pandemic/Coronavirus affected you/people around
  you (in your daily life, personal experiences)?
- How high do you estimate your risk to get infected with the Coronavirus?
- What do you think about the severity of COVID-19 (own risk, risk for people in the household)?
- What are your information resources about COVID-19 and the pandemic in general?
- What do you think about the testing situation in your area (access to tests, relevance of testing)?
- In your view, what role does testing play in the pandemic?
- How accessible do you deem tests?
   What are things that make the access difficult (for you personally or in
   general)?
   What facilitators are there to get tested (for you personally or in general)?
- For asymptomatic individuals: We sent you a test set. Thereupon, you did the test and sent the sample back to us. Our records indicate that you were asymptomatic before taking the test and that you were tested positive for SARS-CoV-2 during the study. Alternative for symptomatic individuals: We sent you a test set. Thereupon, you did the test and sent the sample back to us. Our records indicate that you were tested positive.
  Is that correct?
- Have you ever heard about the gargle test before?
- Can you walk me through your experience from the moment when you were diagnosed? Can you show me how your day went?
- Immediately after receiving the result: What was your initial reaction? Did you speak to someone? How did you organize the quarantine? What did you feel at that moment?
- How did it feel to inform your contact persons? How did contact persons react?
- Can you please walk me through the following days?

How were routines reshaped of your daily life, your household members, your
 community?
 How did your role as [mother/father/employee/employer/...] change?
 What kind of symptoms did you have? Did you develop any symptoms?

- In the long term,
   what did it mean for you to be in quarantine?
   how did your perception of your role as […] change?
   did the way that you perceive yourself change and how?
   how were the people around you affected by your diagnosis?
- What were your expectations of the test/test result before taking the test?
- How did you decide to take the test?

What did you weigh for or against taking the test?

- What consequences did you think the test would have on your life before conducting the test?
- How did you feel before you took the test?
- Did something change in your life In between the time when you sent out the test but had not received the result yet?
- How did you experience the gargle test?
- How would you recommend improving the gargle test and its implementation?
- How would you recommend improving: the information available; the act of doing the gargle test; the shipment of the test; communication of test results?
- What should remain the same?
- Did you ever get tested before with a different test?
- How did you decide to [not] get tested? What and who influenced your decision to get tested?
- If you could choose one test method to test with in the future, which one would you choose?
- Do you trust one test method more than another?
- Would you be willing to get tested in the future?
- In what situation would you be willing to get tested?
- What consequences would regular testing have on your life? Specifically in your role as [mother/father/employee/employer]? Would regular testing have any personal advantages or disadvantages?
- What advantages/disadvantages would regular testing have for society?
- Did the gargle test/this study ultimately have an affect/change on how you see testing/the diagnosis Covid-19?
- How did your opinion of COVID-19, SARS-CoV-2 tests, and quarantine change during the pandemic?
- Is there anything I did not ask you that I should have asked you?
- Would you like to add anything else?
- Do you have any questions that you would like to ask me?
- How was your experience with conducting the interview?
- What would you have liked to be different? Would you have preferred a different way to conduct the interview?
- Did you experience any technical difficulties?
